# The Impacts of Social Marketing Campaigns on Breast Cancer Awareness and Screening: Scoping Review

**DOI:** 10.2196/88568

**Published:** 2026-10-05

**Authors:** Sahar Abduljawad

**Affiliations:** 1 Health Administration And Medical Information, College of Al-Leith Health Sciences, Umm Al-Qura University, Al-Abdiya Campus, Makkah Al-Mukarramah, Makkah, 24381, Saudi Arabia, 966 246811290

**Keywords:** social marketing, social media, breast cancer, awareness, diagnosis, screening

## Abstract

**Background:**

Promotional activities, such as social marketing approaches, are important for raising cancer awareness and increasing breast cancer screening participation. Such interventions are designed to facilitate change and maintain positive health behaviors at both the community and society level. With the growing use of these promotional activities in public health, it is pertinent to map available studies to inform future evaluations and the development of effective interventions.

**Objective:**

This study aimed to review existing studies on how social marketing interventions have been used to promote breast cancer screening and early diagnosis. The specific objectives were to assess the characteristics of social marketing interventions aimed at promoting breast cancer screening, identify the methodological approaches used in evaluating the interventions, identify the outcomes used to measure the impact of social marketing interventions for breast cancer screening, and assess the impacts of social marketing interventions when promoting breast cancer screening.

**Methods:**

A comprehensive literature search was performed in 6 electronic databases to identify qualitative and quantitative evaluations of social marketing interventions targeting breast cancer screening and early diagnosis. Titles and abstracts were reviewed independently by two reviewers, followed by data extraction and verification. The methodological quality was evaluated using the Cochrane Handbook for Systematic Reviews of Interventions. Outcomes were categorized using an adapted version of key performance indicators and metrics related to social media use in health promotion.

**Results:**

Overall, 15 research articles were eligible and analyzed. The 4 types of social marketing interventions identified were health professional and educational strategies, social media or mass media campaigns, community outreach campaigns, and video-based marketing. The predominant intervention source was public health government bodies, followed by regional health services, cancer charities, and educational institutions. Most interventions were designed to influence breast cancer screening behaviors by raising awareness through advertisements, educational interventions, and fundraising activities. Most interventions reached a large audience (>500 people), but participants had mixed awareness and perceptions toward social marketing strategies, and the outcomes were shaped by demographic factors such as cultural backgrounds, age, education, marital status, and occupation. Knowledge gaps, cost concerns, and the lack of physician recommendations also constituted key barriers to screening uptake. Behavior change outcomes were sparingly assessed in most interventions, thereby contributing to limited understanding of mechanisms affecting screening uptake.

**Conclusions:**

Available evidence suggests that social marketing interventions may enhance breast cancer screening and early diagnosis. However, robust evaluations of these interventions and assessment of behavior change outcomes could be performed in future research by using well-established evaluation frameworks and reporting guidelines. Developers and funders of these campaigns should also consider using an evaluation plan before conducting the actual campaign.

## Introduction

Social marketing campaigns (SMCs) are widely accepted approaches and tools for enhancing public awareness of diseases and altering health behaviors [1]. SMCs entail applying marketing principles to facilitate actions in the pursuit of efficient, effective, fair and sustained social transformation, both individually and collectively [1,2]. According to Thompson and Heinberg [3], social marketing applies marketing strategies to develop interventions that enhance individual and societal well-being.

Despite the similarity to conventional commercial marketing in terms of strategic approach and goal [4], SMCs emphasize voluntary behavioral change and broader benefits directed toward the community and society [1,5]. By shifting from simply providing information, SMCs assist66 with designing a comprehensive, target-centric experience that makes health behaviors highly desirable and easily adopted [1]. Although the product of commercial marketing is specific, social marketing products or services are mostly invisible and conceptual.

Social and health interventions can be enabled by using advertising techniques that promote related commercial products [1]. Typical examples of social marketing include disaster relief and recovery [6], organ donation [7], campaigns promoting recycling [8], and health promotion programs delivered via social media platforms . Social media has become extremely popular, estimated to be used by more than one-half of the global population [[9]. Hence, apart from being widely used for public engagement, it has increasingly extended into social marketing strategies for driving public health interventions [10]. Such integrations can shape behavior by targeting emotional and cognitive responses, triggering discussions, and altering social norms. In addition, the combination of social marketing and social media approaches allows rapid dissemination of health information, at a low cost, to a large audience of people across wide geographical locations [11].

In this article, the term “intervention” refers to a spectrum of activities ranging from national awareness-raising public health campaigns to targeted activities that use social marketing approaches to deliver information to specific audiences, specifically to enhance breast cancer screening decisions and uptake. The effectiveness of these social marketing strategies may differ depending on the approach used, whether official, emotive, or persuasive. The source of the message is pivotal in its persuasiveness, as sources that hold higher status and are familiar to the recipient are more likely to be recalled and internalized [12].

Accumulated evidence suggests that raising awareness and promoting the prevention of cancers has several challenges. The barriers to cancer screening uptake among susceptible individuals can be resolved via interventions that educate people about the benefits of early detection and screening [5,10]. Promotional activities, such as social marketing approaches, are critical for raising cancer awareness and likely to increase screening participation among high-risk persons, thereby reducing related morbidity and mortality rates [10]. The goal of promotional activities is to facilitate change and maintain positive health behaviors at the community and society levels.

In terms of breast cancer, the level and impact of social marketing strategies on screening decisions and uptake are underreported despite the extensive barriers to screening and adherence in vulnerable populations [13,14]. Nevertheless, empirical studies have demonstrated the effects of awareness programs on health behaviors for other diseases and disorders. Examples include the strong empirical evidence on the reduction of smoking prevalence and promotion of smoking cessation following mass media campaigns [15]. It is estimated that public health campaigns designed to address coronary heart disease have led to a 30% reduction in cholesterol in the past 3 decades [16].

Given these social marketing effects, the potential benefits of awareness raising and promotional activities on breast cancer screening should not be underestimated, thus warranting further investigation. Many scholars have attempted to explore the effectiveness of promotional campaigns in raising awareness and motivating screening for breast cancer and other cancers [17,18]. The outcomes from these studies have varied. The perceived credibility of breast cancer information is shaped by factors such as education, socioeconomic status, age, health attitudes, health status, and cultural background [19,20].

Moreover, despite the increasing advocacy for promotional campaigns, there is little evidence to support their efficacy in improving breast cancer screening and early diagnosis. Review publications describing the various approaches in which social marketing strategies are used to promote breast cancer screening and how such interventions might foster behavior change are lacking. Previous scoping and systematic reviews looking at a range of interventions for breast cancer screening focused on social media-based and mobile health (mHealth) technology [5]. However, the use of social marketing interventions for public health is rapidly evolving, reflecting the need for a broader mapping of available studies to inform future evaluations and the development of effective interventions.

This study aimed to review existing studies on how social marketing interventions have been used to promote breast cancer screening and early diagnosis. The specific objectives were to assess (1) the characteristics of social marketing interventions aimed at promoting breast cancer screening, including methodological approaches and outcomes measures, and (2) the impacts of social marketing interventions on promoting breast cancer screening. By emphasizing nonclinical initiatives, this study reviewed SMCs with the potential to be implemented at the community level to promote breast cancer screening and preventive behaviors.

## Methods

### Study Design

This scoping review was conducted in accordance with PRISMA-ScR (Preferred Reporting Items for Systematic Reviews and Meta-Analyses Extension for Scoping Reviews) [21] and guided by the scoping review framework by Arksey and O’Malley [22]. The review focused on the use of social marketing interventions for promoting breast cancer screening and positive behavioral changes in vulnerable populations. The review was designed to address the following research questions: (1) What are the characteristics of social marketing interventions that aim to promote cancer screening? (2) What methodological approaches have been used to evaluate the interventions? (3) What are the outcomes used to measure the impact of interventions? and (4) What are the impacts of social marketing interventions in promoting breast cancer screening?

### Search Strategy and Search Terms

A comprehensive literature search was conducted in 6 electronic databases: Web of Science, PubMed, EBSCOhost (via CINAHL), MEDLINE, PsycINFO, and Scopus. The search included studies using quantitative, qualitative, and mixed methods designs to ensure a broad retrieval of relevant literature. To focus on recent advancements and growing research on social marketing strategies for breast cancer screening uptake and preventive behaviors, the search was limited to articles published between 2010 and 2025.

The literature search was performed from August 2025 to October 2025. In addition to the database search, snowballing techniques were applied, including backward reference checking (screening reference lists of selected articles) and forward reference searching using the “cited by” function in Google Scholar. These manual searches were performed to retrieve possible articles that might be missed from the electronic databases. Aligning with the research objectives, the following 6 key search components were identified: (1) breast cancer; (2) screening or testing; (3) SMC, promotional activities, or advertisement; (4) social media, mass media, or mass communication; (5) intention to screen or test; and (6) behavior change or preventive behavior.

Alternative keywords were used for each component and combined using the Boolean operators “OR” within categories and “AND” between categories. Boolean operators were not applied in Google Scholar. Additionally, a truncation symbol (*) was used where applicable to capture word variations, as detailed in Tables 1 and 2.

**Table 1.** Search strategy for the literature review in the following databases: Web of Science, PubMed, EBSCOhost (CINAHL Complete), Scopus, MEDLINE, and PsycINFO.

Main keywords	Search strings
Breast cancer	“Breast cancer” OR “Breast cancer patients” OR “Women with Breast Cancer”
Screening, testing	“Breast cancer screening” OR “Breast cancer testing” OR “Screening” OR “Testing
Social marketing campaign, promotional activities, advertisement	“Social marketing” OR “Campaign” OR “Promotion” OR “Advert”
Social media, mass media, mass communication	“Social media” OR “Mass media” OR “Mass communication” OR “Communication”
Intention to screen, test	“Intention to screen” OR “Intention to test”
Behavior change, preventive behavior	“Behavior change” OR “Preventive behavior”

**Table 2.** Specific search strings used in each database for the literature search.

Search number	Specific search query and string	Search engine(s)
1	((Breast cancer screening OR Breast cancer test OR Breast cancer testing OR intent to test)) AND (Social marketing OR social marketing campaign OR mass communication OR mass media OR mass media campaign)	Scopus
2	((Breast cancer screening [Title/Abstract] OR Breast cancer test[Title/Abstract] OR Breast cancer testing[Title/Abstract] OR intent to test[Title/Abstract])) AND (Social marketing campaign [Title/ Abstract] OR mass communication [Title/Abstract] OR mass media campaign [Title/Abstract])	PubMed
3	((Breast cancer screening [Title/Abstract] OR Breast cancer test [Title/Abstract] OR Breast cancer testing [Title/Abstract] OR intent to test [Title/Abstract])) AND (Social marketing [Title/Abstract] OR social marketing campaign [Title/Abstract] OR mass communication [Title/Abstract] OR mass media [Title/Abstract] OR mass media campaign [Title/Abstract])	Web of Science
4	((Breast cancer screening[Title/Abstract] OR Breast cancer test[Title/Abstract] OR Breast cancer testing[Title/Abstract] OR intent to test[Title/Abstract])) AND (Social marketing[Title/Abstract] OR social marketing campaign[Title/Abstract] OR mass communication[Title/Abstract] OR mass media[Title/Abstract] OR mass media campaign[Title/Abstract] OR exchange theory[Title/ Abstract] OR audience segmentation[Title/Abstract]	EBSCOhost (CINAHL Complete), MEDLINE, PsycINFO

### Study Selection and Screening

The study included patients with breast cancer and those at risk who had participated in interventions involving any type of social marketing strategy, such as health promotion activities and community awareness programs. Titles, abstracts, and full-text articles were screened using PDF content to ensure alignment with the study objectives. Selected articles were subsequently stored in a Zotero database for reference management.

### Eligibility Criteria

Articles were included if they met the specific criteria outlined in Table 3, which included the language and publication period, study design, intervention and outcomes, and target population. Meanwhile, studies not fulfilling the criteria listed in Table 3, not published in English, not focusing on breast cancer patients, or not involving cancer survivors or health professionals were excluded. Review articles, online materials, conference proceedings, nonoriginal articles, and articles that evaluated static internet pages, such as blog posts, were also excluded.

**Table 3.** Criteria and description considered for eligible studies.

Criterion	Description
Language and publication period	Original research articles or short communications written in English, published between 2011 and 2024
Study design	Interventional studies, including quantitative, qualitative, and mixed methods
Intervention and outcomes	Studies had to examine social marketing interventions/campaigns/programs and address at least one of the following outcomes: breast cancer screening, early diagnosis, preventive practices, or help-seeking for potential breast cancer symptoms.
Target population	Studies had to focus on women with breast cancer or high-risk populations.

### Screening Process and Data Extraction

Title and abstract screening were conducted by 2 researchers, followed by full-text assessment for eligibility. Disagreements were resolved through consensus, with a third reviewer serving as a mediator. An interrater reliability analysis was performed for title and abstract screening, and an acceptable level of agreement was achieved (Cohen α=0.72). Data extraction was conducted independently by 2 research assistants to ensure accuracy and consistency. The lead researcher predefined the key data points to be recorded. A structured data extraction form was used, capturing the following details: authors’ names, research objectives, study location and design, population of interest, interventions applied, and main findings. Any discrepancies during data extraction were resolved using the same consensus approach described in the screening process, with a third reviewer providing mediation when necessary.

### Assessment of Risk of Bias

Although quality appraisal is not always required in scoping reviews due to the diversity of study designs [23], this review used a risk of bias assessment given its specific focus on social marketing strategies or programs for women with breast cancer and high-risk groups. The methodological quality of each study was evaluated using the Cochrane Handbook for Systematic Reviews of Interventions [24]. The assessment considered 5 key domains: sequence generation, allocation concealment, adjustment for confounders, blinding, completeness of data, and selective reporting.

### Data Synthesis

The extracted data and outcomes from the reviewed studies are shown in Table 3. We categorized the outcomes using an adapted version of key performance indicators and metrics related to social media use in health promotion [25]. The main aspects include insights, exposure and reach, and various levels of engagement. Information on the design, theoretical foundation, and delivery methods of interventions was also extracted from the reviewed studies. Furthermore, information regarding the potential mechanisms by which social marketing interventions shape behavior change was gathered by using the behavior change technique (BCT) taxonomy described by Michie et al [26].

The extracted data were analyzed using a narrative approach and thematic analysis, as recommended for scoping reviews. An inductive approach was used to identify and synthesize key themes based on the main findings from each study. The data synthesis process involved familiarization and coding, performed independently by 2 research assistants. The next step entailed developing the themes, whereby systematic coding was used to generate overarching themes. The last stage was validation and consensus, in which all the themes were reviewed by the authors to ensure consistency with the extracted data. Discrepancies were also resolved through discussion. All data management and analysis procedures were conducted using Excel (Microsoft Corp).

## Results

### Search Results

The literature search retrieved 724 articles from 6 databases. More than one-half (572/724, 79%) of the results were removed before screening, as 450 were identified as duplicates and 122 records were marked as ineligible. Upon title and abstract screening, 69 records were excluded. In the final stage, 15 of the 58 articles were eligible and included in the qualitative and thematic analysis. The reasons for excluding 43 full-text articles are outlined in Figure 1.

**Figure 1.**
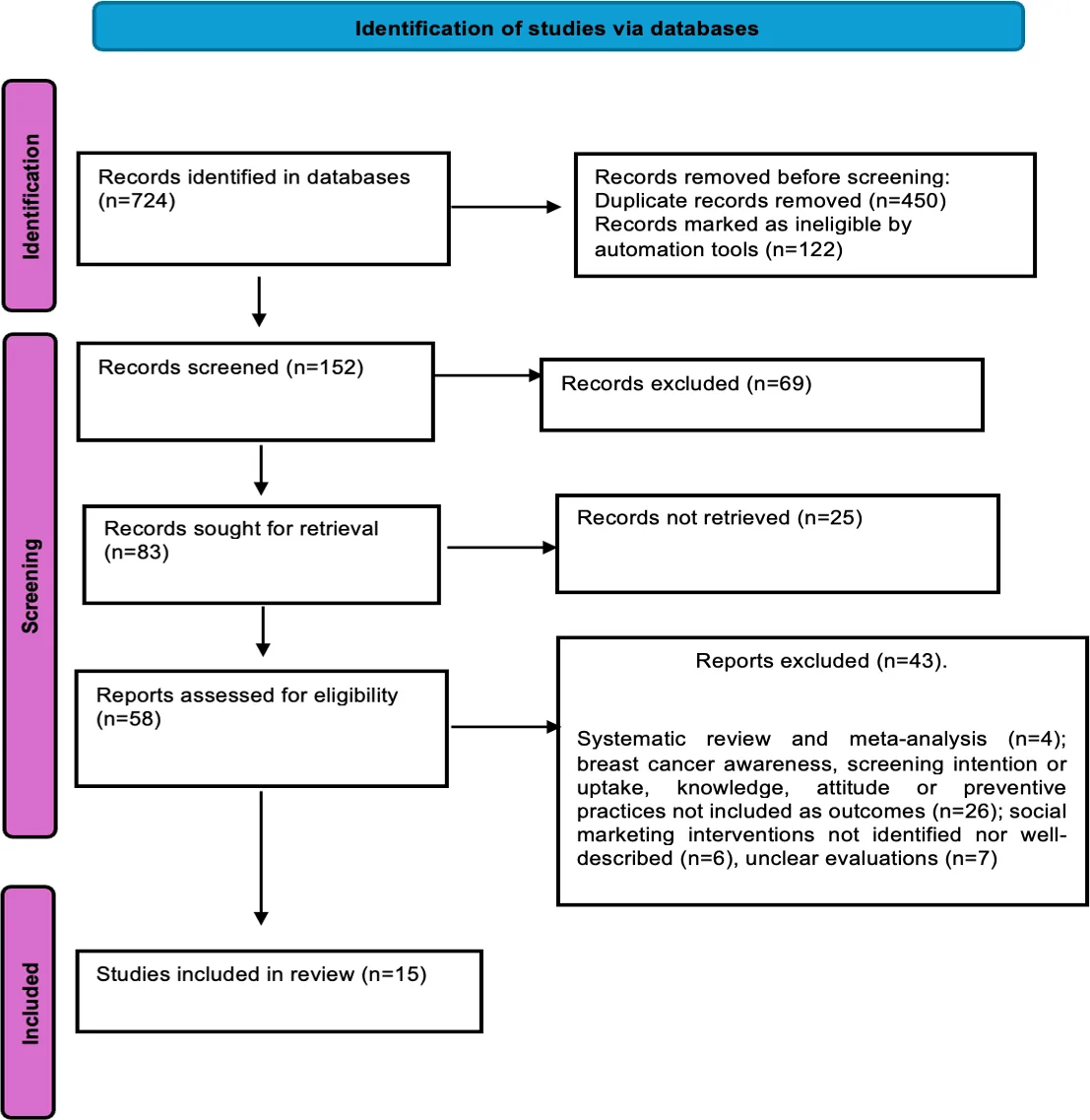
Flowchart of the scoping review literature search and screening process.

### Studies and Participants’ Characteristics

Table 4 outlines the characteristics of the reviewed studies. All the articles were original studies published in peer-reviewed journals. Most of the studies were conducted in the United Kingdom (n=4), and 1 each was conducted in Greece, Malaysia, Romania, Saudi Arabia, the United Arab Emirates, India, the United States, Israel, Turkey, China, and South Korea. In terms of study design, 4 studies were either single or multicenter randomized controlled trials (RCTs), 4 were quasi-experimental designs, 4 comprised a combination of cross-sectional surveys and pre- and posttest designs, 2 were case studies, and 1 article involved content analysis and in-depth interviews.

**Table 4.** Characteristics and main findings from the 15 studies on social marketing campaigns.

Search number	Reference	Objective	Study design, sample population	Sample population	Mode of delivery	Specific instruments and interventions	Main findings
1	[18]	To examine the effects of a TV-led breast cancer mass media campaign on the awareness of breast cancer–related factors and intention to engage in preventive behaviors	3 campaigns performed over 2 weeks: precampaign, followed immediately by campaign wave 1 and campaign wave 2 in the northeast of England	572 in the precampaign; 576 in wave 1; 552 in wave 2	Cancer charity	TV advertisements showing the connection between breast cancer and alcohol consumption, with a voiceover communicating the various types of cancer caused by alcohol; target online audience also reached via advertisements shown on promoted posts on Facebook	Awareness of breast cancer and readiness to engage in preventive behaviors increased significantly postcampaign (45% increment) compared with the precampaign level, especially after wave 2. Awareness of alcohol as a cancer risk factor and support for related policies increased significantly between surveys.
2	[27]	Effects of an early presentation (PEP^a^) intervention on breast cancer knowledge, skills, and motivation for early presentation into care among older women	Randomized controlled trial	867 women 67‐70 years old attending a breast cancer screening program	Regional health services	PEP intervention using a 10-minute interaction with a radiographer	At 2-year follow-up, the intervention increased the proportion of participants who were aware of breast cancer and ready to perform early screening compared with those in usual care (21% versus 6%; OR^b^ 8.1, 95% CI 2.7%‐25.0%)
3	[28]	Phase 2 assessment of the impact of a brief health professional–delivered intervention on breast cancer awareness and prevention intention among older women	Multicenter clinical trial; mammographers trained to deliver a brief health intervention for older women (n=495) in 4 screening centers, with 1 used as a control (no intervention); breast cancer awareness and prevention intention assessed at baseline and 1 month postintervention	875 older women (no intervention)	Regional health services	The intervention was based on the English National Health Service Breast Screening Program, designed to equip women to present early with breast symptoms, as a critical aspect of the concluding invited mammogram at an estimated age of 70 years.	A total of 27 mammographers were trained. Overall, breast cancer awareness increased 7-fold at 1 month in older women exposed to the intervention relative to a 2-fold increase in the control group (OR 15.2, 95% CI 10.0 to 23.2).
4	[29]	To identify the breast cancer screening advertisements preferred by women at risk of breast cancer	A hybrid study design, comprising content analysis and repertory grid interviews; first approach: identified pertinent messages mostly promoted in breast cancer screening advertisements; interview aimed to elucidate women’s perceptions regarding the messages; 13 different breast cancer screening advertisements presented to assess level of authoritativeness, credibility, emotional appeal, and persuasiveness; conducted in Greece	32 women	Charity body, celebrities	13 breast cancer screening advertisements	Some women found some of the breast cancer screening promotional messages, including award-winning advertisements, offensive and failed to connect with the visual design of these adverts. Respondents were more motivated to perform screening when exposed to positive advertisements, capturing reliable data on breast cancer data and endorsements from celebrities who genuinely support the cause.
5	[30]	To assess the effects of a mass media campaign on breast cancer symptoms awareness and screening uptake	Pre and posttest experimental study (quasi-experimental design) in Malaysia	676 women aged >40 years from randomly selected households (intervention group)	Public health government bodies	A culturally adapted mass media campaign (TV, radio, print media, and social media); outcomes: awareness of breast cancer symptoms, self-efficacy to identify breast cancer symptoms and clinical outcomes, and campaign reach; mammogram screening and clinical breast examination data collected from hospitals and clinics	At least one campaign material was identified by most participants (65.2%); Chinese women (OR 0.25, 95% CI 0.15 to 0.40) and those aged >70 years (OR 0.47, 95% CI 0.23 to 0.94) demonstrated lower odds of being reached during the campaign relative to Malays and younger women; higher level of postcampaign awareness was observed among participants who recognized the campaign compared with nonrecognizers, especially for primary symptoms such as discharge or bleeding from nipple (79.7% vs 55.3%) and “a lump or thickening in your breast” (88.9% vs 62.1%); none of the demographic variables influenced improvement in symptom awareness scores.
6	[31]	To explore the impact of social media awareness campaigns on the early detection of breast cancer	Online survey conducted after periodic campaigns in Romania	1945 participants selected from a Facebook community (intervention) and 289 participants in a control group	Public health government bodies	Intervention comprising of periodic campaigns on the significance of breast cancer screening for prevention and early diagnosis	Intervention group had significant improvement in breast cancer screening awareness and preventive practices relative to the control group.
7	[14]	To assess the efficacy of an institutional breast cancer health campaign on the knowledge, attitudes, and practices of female employees	Cross-sectional descriptive study. in Saudi Arabia	290 female workers from various university departments	University	A group-based breast cancer awareness campaign, supported with posters, brochures, lectures and advertisements, followed by an online self-administered questionnaire	Knowledge and positive attitudes toward breast cancer increased significantly postintervention; participants who had previously attended any prior breast cancer health campaign scored higher knowledge (mean 7.45, SD 0.98) and attitude (mean 5.30, SD1.48) scores than those who had never attended (knowledge: mean 6.10, SD 1.10; attitude: mean 4.10, SD 1.41); to facilitate screening behavior, counseling services and the promotion of existing breast cancer screening are necessary; diverse, culturally sensitive strategies tailored to younger and older women are recommended in future interventions.
8	[32]	To ascertain the effects of breast cancer awareness programs on the use and uptake of breast screening among women in the United Arab Emirates	Cross-sectional study in the United Arab Emirates using a content-validated interviewer-administered questionnaire	959 women aged ≥20 years	University	—^c^	One-third of the participants (31.7%) had attended awareness programs, and attendance was influenced by younger age, higher education, and being single (*P*<.001); screening was performed by 38.3% of participants who had ever attended any breast cancer awareness programs than nonattendees (13.3%; *P*<.001); important barriers included knowledge gaps, cost concerns, and the lack of physician recommendations.
9	[33]	To evaluate the impact of a video-based intervention on the knowledge of breast cancer risk factors, symptoms, and attitudes toward screening practices	A quasi-experimental design in Bihar, India	267 rural-dwelling women	Regional health services	Intervention entailing serial exposure to validated YouTube Hindi videos focusing on educating respondents on breast cancer, changing attitudes toward screening methods, and encouraging healthy prevention practices	Knowledge level of risk factors, symptoms, and breast cancer screening increased significantly (100%) at 3 months post-intervention. Attitude towards breast self-examination improved significantly in 58% of women, 16.5% for clinical breast examination, and 9.7% for mammogram. Breast self-examination practices improved significantly in 60% of women. Associated factors of breast cancer awareness include education, age, and occupation.
10	[34]	Exploring participatory social marketing principles for culturally adapted evidence-based cancer screening programs at the community level	Case studies in New York City, United States	Muslim women	Public health government bodies	The case entailed a culturally adapted program to increase breast and cervical cancer screening among a multiracial and ethnic population.	The case study revealed the benefits of integrating social marketing and CBPR^d^ approaches as key strategies for the development of public health campaigns; they were effective in reaching and influencing health behaviors among vulnerable populations in the community, particularly the socially marginalized.
11	[35]	To explore the effects of peer trainer intervention on awareness of early detection and diagnosis, use of early diagnosis methods, and screening centers among women in Turkey	Quasi-experimental design in Turkey	40 women (>40 years old) participated as peer educators, with 20 selected as principal peer educators; 200 women received the education	Public health government bodies	Peer trainers educated their peers, followed by arranging a mammography appointment for women who were interested in taking the test	Participants demonstrated positive changes in health beliefs and breast self-examination knowledge; of the ~20% (n=1040) who did get mammograms, 8% were positive for breast cancer.
12	[36]	To assess the effects of a culturally based intervention program on adherence to breast cancer screening	Randomized controlled trial with a 3-month follow-up survey in Israel	589 ultra-Orthodox Jewish or Arab women	Public health government bodies	Individually tailored intervention based on each participant’s degree of adherence, knowledge, and beliefs	In both groups, the performance of breast awareness practice and clinical breast examination was associated with a lower level of religious beliefs and a better understanding of the importance of screenings.
13	[37]	Impact of mass social events on awareness of breast and cervical health among Black women	Case studies, with an average attendance time >1 hour at each event in the United Kingdom	>450 attendees at each event	Public health government bodies	Series of virtual educational and cancer screening events based on the motive to build a breast cancer resource hub for Black women (Breast/Best Health for Black Women [BHFBW]); the program was a collaboration between the research institution and relevant community organizations; the intervention was designed to target the individual needs of Black women.	Most respondents (>85%) perceived that the event assisted them with feeling supported; about 46-48 women underwent a screening mammography during the events in 2022 and 2023; in both periods, >9% of participants affirmed they were likely to go for a mammogram when necessary.
14	[38]	To determine the influence of fear appeal messages available in media outlets on intention to perform breast self-examination among women in China	2-by-2 factorial experiment in China	488 women aged 25-50 years	Public health government bodies	Provision of messages about the risks and precautions of breast cancer across different media outlets and the intention to perform breast cancer self-examination using the EPPM^e^	Threat and efficacy had significant main and interaction effects on intention to perform breast cancer self-examination; no significant difference was detected in terms of the effectiveness of fear appeal messages between traditional and social media outlets.
15	[39]	To promote screening mammography and correct breast cancer myths among women living in an urban community in Korea	A multicity, multicomponent community intervention trial, with surveys at baseline and 7 months later in South Korea	480 women	Public health government bodies	The intervention city was equipped with a community outreach and clinic and pharmacy-based in-reach strategies; the community campaign emphasized the efficacy of screening mammography for detecting masses that are not reachable; messages were tailored to correct myths about breast cancer and screening mammography.	A 20.4%-point decrease in the myths regarding the association between breast size and cancer, a 19%-point decrease in myths about the cost of mammography, and a 14% increase in the intention to perform screening mammography. In the intervention city, about a 23% increase in the proportion of participants at the action stage of the transtheoretical model, but such improvement was either lacking or inconsistent in the control city.

a PEP: Promoting Early Presentation.

b OR: odds ratio.

c Not available.

d CBPR: community-based participatory research.

e EPPM: extended parallel process model.

The sample size of participants in the studies ranged from 32 to 1945 with a total of 10,287 women enrolled in the intervention groups and 1164 in the control groups accross the 15 studies. The participants’ ages were presented in 8 studies, with a minimum age of ≥20 years to a maximum age of 70 years old. Most studies used >40 years old as the eligibility criterion.

### Characteristics and Methodological Approaches Used in Social Marketing Interventions

This section presents findings regarding the first objective, focusing on the characteristics of social marketing interventions in the context of breast cancer screening. Specifically, the results are described based on intervention source and types; underpinning theories; insights, exposure, and reach; and outcome measures.

#### Intervention Source and Types

Public health government bodies were the predominant intervention source (8/15; 53%), followed by regional health services (3/15, 20%), cancer charities (2/15, 13%) and a university (2/15, 13%). The interventions can also be broadly divided into national (n=10) and regional (n=5) breast cancer awareness campaigns, as well as targeted (n=9) and untargeted types (n=6). National breast cancer awareness campaigns refer to those performed to enhance breast cancer screening and awareness among the general public. Meanwhile, regional types focus on a specific region or community. Targeted interventions encompassed those that used well-defined activities to disseminate breast cancer screening and awareness information to specific groups, whereas untargeted types refer to interventions delivered to the general public without specifically focusing on breast cancer.

Overall, 4 types of social marketing interventions were identified: (1) video-based marketing (n=2) [18,33], (2) health professional and educational strategies (n=5) [14,15,27,28,32], (3) community outreach campaigns (n=3) [34,36,39], and (4) social media or mass media campaigns (n=5) [30,31,37,38,40]. Thus, the majority of studies involved social marketing strategies incorporating health professional and educational approaches, followed by social media and outreach campaigns.

#### Behavior Change Theories Used in Interventions

Five studies provided insight into the theories that informed the development and implementation of the intervention. The predominant models considered were the health belief model [30,35,36], the transtheoretical model [39], and the extended parallel process model of persuasion [38].

All interventions were designed to influence breast cancer screening behaviors, such as improving attendance at cancer screening, performing self-examination, and adhering to recommended prevention practices. This was achieved by disseminating information on breast cancer screening and raising awareness through advertisements, educational interventions, and promotional or fundraising activities.

The interventions shaped behavior change by informing participants or vulnerable people regarding the health consequences of breast cancer, specifically by providing videos, posters, advertisements, brochures, modules, and links to cancer charity and public health websites to access information about screening, risk factors, and symptoms. The interventions delivered information from well-established and credible sources, such as cancer charities, national and regional government health organizations, and university or institutional bodies, but certain information from untrusted individuals, users, or celebrities was deemed less credible [18]. Interventions that used social comparisons, individuals’ approval, and social support to motivate cancer screening were reported in 4 studies. Examples included sharing messages of support from organizations, the public, cancer survivors, charities, and celebrities [18,31,35,36,39].

#### Insights, Exposure, and Reach

Different exposures were used in line with the type of intervention. Some of the studies provided detailed information on the exposure and the reach achieved by the social marketing interventions. One of the interventions with the largest reach involved 2 waves of a social campaign performed over 2 weeks with 1200 participants in the northeast of England. The target online audience was also reached via advertisements shown on promoted posts in Facebook [18]. A similarly large audience was reached by studies conducted in the United Arab Emirates [32], reaching 959 women aged ≥20 years; Turkey [35] with 20 trained peer educators and each educating approximately 200 women; and more than 450 attendees in a series of campaign events organized to build a breast cancer resource hub for Black women in the United Kingdom [37]. In Malaysia, about two-thirds of the participants identified at least one of the campaign materials used for the intervention, and the probability of being reached during the campaign was lower among Chinese women (odds ratio [OR] 0.25, 95% CI 0.15 to 0.40) and those aged >70 years (OR 0.47, 95% CI 0.23 to 0.94) relative to Malays and younger women [30].

A few studies shared insights into participants’ views on the acceptance of social marketing strategies as an approach to delivering breast cancer awareness and screening information [18,37]. However, 1 study revealed that some women perceived that some breast cancer screening promotional messages, including award-winning advertisements, were offensive and did not represent the benefits or messages portrayed in the advertisements [29].

#### Outcome Measures

Descriptively, 5 studies reported outcomes relating to breast cancer awareness [18,27,28,30,40]; 6 studies reported outcomes for early cancer screening and preventive behaviors [27,31,35,38-40]; 4 studies reported outcomes related to perceptions regarding social marketing strategies for prompt cancer screening [29,30,34,37]; 6 studies reported outcomes regarding knowledge, attitudes, and practices toward breast cancer screening [14,32,33,35,36,39]; and 4 studies reported on the predictors of breast cancer awareness and screening [14].

### Impacts of Social Marketing Interventions

#### Breast Cancer Awareness

Martin et al [18] used TV advertisements to explore the relationship between breast cancer and alcohol consumption, supported with voice notes and Facebook posts on different types of cancer caused by consuming alcohol. The intervention led to a significant postcampaign increase (45% increase) in breast cancer awareness compared with the precampaign level. In Malaysia and Romania, culturally adapted and mass media campaigns (TV, radio, print media, and social media) led to significantly higher levels of awareness among participants who recognized the campaign than nonrecognizers [30]. However, the true effects of these social marketing interventions on breast cancer awareness remain uncertain given the methodological limitations, particularly the use of a 1-group pre and postdesign and the lack of underpinning theories.

Meanwhile, more reliable findings were observed in 2 RCTs [27,28]. A multicomponent intervention, comprising Promoting Early Presentation (PEP) and a 10-minute interaction with a radiographer, improved breast cancer awareness by 2-fold (OR 8.1, 95% CI 2.7%‐25.0%) compared with participants in usual care at the 2-year follow-up [27]. A similar intervention based on the English National Health Service Breast Screening Program for older women (approximately 70 years old) was associated with a 7-fold increase in breast cancer awareness (OR 15.2, 95% CI 10.0 to 23.2) relative to a 2-fold increase in the control group. These results suggest that a multicomponent intervention incorporating SMCs and health promotional activities is more effective at upscaling breast cancer awareness. However, clinical trials comparing these various approaches are required to test the hypothesis.

#### Breast Cancer Screening and Preventive Behaviors

The significant effects of SMCs on early breast cancer screening and preventive behaviors were revealed in 3 intervention trials [27,38,39]. The PEP intervention investigated by Forbes et al [27] among vulnerable women in the United Kingdom led to an 8-fold increase in participants’ readiness to perform early screening. The intention to perform breast cancer self-examination also improved following the provision of messages about the risks and precautions of breast cancer across different media outlets [38]. Likewise, community outreach and clinic and pharmacy-based in-reach strategies led to a 14% and 23% increase, respectively, in the intention to perform screening mammography and the proportion of participants at the action stage [39].

A few studies were designed to shape participants’ perceptions and positive behavioral change toward breast cancer screening via community outreach campaigns [34], social media or mass media campaigns [29,37], and health professional and educational strategies [14,32]. Yfantidou and Skandali [29] conducted a qualitative interventional study by presenting breast cancer screening advertisements to 13 participants, followed by interviews to capture their perspectives on the promotional activities. Some of the breast cancer screening and promotional activities were considered offensive and failed to connect with the visual designs; however, the participants were motivated to perform screening when exposed to positive advertisements capturing reliable data on breast cancer and endorsements from highly respected celebrities. Williams et al [37] used a similar intervention involving a series of virtual educational and cancer screening events used to build a breast cancer resource hub for Black women. As a result, most participants (>85%) perceived the SMC to be very helpful and supportive, leading to a 9% increase in the proportion of those likely to visit health care centers for mammogram screening.

Some of the studies in this category, particularly those involving health professional and educational strategies [32,35,37] and community outreach campaigns [36,39], highlighted a positive relationship between improved knowledge and a positive perception toward breast cancer screening. The video-based social marketing used by Sinha and Sharma [33] in a regional study in India also reported significant improvements in participants’ knowledge of risk factors, symptoms, and breast cancer screening, leading to positive attitudes toward breast self-examination. Direct comparisons between these interventions is challenging given the different approaches. More importantly, the evidence from these studies is weak given the qualitative or quasi-experimental design and the use of an online survey for follow-up assessment [35].

### Factors Influencing the Primary Outcomes

The factors influencing breast cancer screening awareness were investigated by 4 articles. Alomairy et al [14] found that cultural backgrounds shaped younger and older women’s attitudes toward breast cancer screening services. Abdulla et al [32] and Sinha and Sharma [33] reported that younger age, higher education, occupation, and being single improved the likelihood of attending breast cancer awareness programs, with knowledge gaps, cost concerns, and the lack of physician recommendations constituting the major barriers. The performance of breast awareness practices and clinical breast examinations was associated with having a lower level of religious beliefs and better understanding of the importance of screenings [36]. In contrast, none of the demographic variables influenced improvement in symptom awareness scores among Malaysian women who were exposed to a culturally adapted and mass media campaign (TV, radio, print media, and social media) [30]. These findings reflect the multifactorial nature of breast cancer screening behaviors and the need for tailored social marketing interventions.

## Discussion

### Principal Findings

Most articles on social marketing interventions assessed breast cancer awareness and screening intentions using a mixture of experimental and observational study designs to measure exposure, reach, and impacts on investigated outcomes. A few studies revealed that national and regional breast cancer awareness campaigns and targeted interventions might improve cancer awareness, screening intentions, and uptake. The available evidence also suggests that exposure, reach, and engagement attained by these interventions differed by age, gender, educational level, and users’ ethnicity.

Although several reviews have been published on interventions to address cancer screening and prompt diagnosis, to the best of our knowledge, this scoping review is the first attempt to focus on social marketing interventions to improve breast cancer screening, awareness, early diagnosis, and presentation to care. The findings add to the existing knowledge pool by reflecting the data paucity and robust clinical trials exploring the level of engagement, mechanisms of behavior change, and attendance at screenings specifically designed for women with breast cancer and high-risk groups. It is difficult to attain high engagement levels given the challenge with accessing timely observational data on breast cancer screening attendance [40,41]. This is further complicated by the fact that social marketing interventions are usually designed without provision for evaluation and impact metrics [42]. A similar situation was observed in the scoping review conducted by Plackett et al [5], whereby the authors commented, based on expert validation of emerging themes, that campaigns are designed rapidly without room for evaluation.

Immediate changes are difficult to measure since cancer screening and early diagnosis are long-term goals, which is in sharp contrast to the primary design of SMCs. More comprehensive evaluations that reflect outcomes of behavior change and engagement could be achieved by developing an evaluation framework that integrates components of behavior change theories [43]. Nevertheless, it is difficult to equivocally demonstrate that a specific campaign is primarily responsible for a change in behavior or outcome, in addition to the organizations’ limited resources and time to perform evaluations. Such assessments could, however, be conducted in campaigns involving collaboration with public health and institutional bodies.

In this review, we attempted to use the BCT taxonomy to delineate the various BCTs used in social marketing interventions to shape participants’ health behaviors, including social support, incentives, and equipping vulnerable populations with health information. The health belief theory was the framework predominantly used to develop the investigated interventions. According to BCTs, providing actionable health information about breast cancer symptoms and risk factors could have stronger and direct positive effects on behavior change. The BCTs used in breast cancer campaigns, such as sharing personal stories, could help create social support and influence public opinion, thereby encouraging help-seeking behaviors, increasing screening uptake, and improving health outcomes. Future interventions could explore these assumptions, particularly how national and regional social marketing strategies might facilitate positive behavior change and the most important messages required in such interventions.

This scoping review revealed the degree to which social marketing interventions addressed inequalities in breast cancer screening, awareness, and early diagnosis. The available evidence suggests a low reach and engagement with ethnic minorities [1,30], which is mainly associated with limited access to breast cancer screening, stigma, and low engagement with social media and SMCs [44,45]. This inequality is further exacerbated when the information is not culturally adapted [46]. The low uptake of screening and awareness may also stem from the lack of campaign role models that are highly respected and resonate with ethnic minorities [18].

Inequalities in the use and impacts of SMCs were also identified in terms of age, educational level, marital status, and occupation. Younger age, higher education, being employed, and being single improved the odds of exposure to breast cancer awareness programs and perceived benefits toward screening services [14]. These results align with recent data on social marketing and media use in the United Kingdom and high-income countries by age and gender [47]. The finding underscores the need for specific campaign strategies preferred by target users and those more likely to facilitate broad engagement.

Future interventions involving social marketing approaches should assess inequalities in exposure, reach, and engagement, as well as consider the effectiveness directly in relation to the target groups. Designing interventions toward populations with a disproportionate disease burden could address health inequalities observed in breast cancer screening and early diagnosis. This could be achieved by shifting from broad, “one-size-fits-all” approaches to audience-segmented interventions that effectively tackle systemic barriers. Social marketing strategies involving mass and social media can shift the “product” from fear-inducing to capacity-building, relatable, and manageable actions [48]. Educational-related barriers could be addressed by incorporating language and cultural nuances, partnering with community leaders, and designing messages that align with cultural beliefs and mitigating local cancer myths [49].

Meanwhile, inequities introduced by unemployment could be tackling systemic and hidden costs. Examples include campaigns that promote screenings related to tangible solutions, such as free and accessible mammography centers in local communities. These marginalized groups could also be further reached by expanding distribution channels beyond digital spaces, focusing on physical and trusted touchpoints [48]. Other possible channels to address the aforementioned inequities include leveraging trusted intermediaries to deliver face-to-face messaging and engaging the support ecosystem while considering the role of family structures in health care decisions [5].

Based on the syntheses of the 15 articles, most SMCs and interventions had significant impacts on breast cancer screening awareness and preventive behaviors. Overall, the findings are consistent with previous reviews on the use of social marketing strategies in HIV testing and related outcomes [49], whereby a consistent pattern of positive results were observed. Nevertheless, effectiveness seems to differ depending on specific elements used in the intervention, such as mass media, social media, and recognizable figures. Methodological limitations also need to be considered when interpreting the findings.

For instance, 2 RCTs [27,28] incorporating SMCs and health promotional activities led to significant improvement in upscaling breast cancer awareness, with a large effect size. These promising results may stem from the efficacy of such campaigns at enabling the transition from mere information to behavioral change. By integrating health promotional activities such as culturally tailored education, the intervention directly addresses psychosocial barriers, strengthens self-efficacy, and encourages proactive health actions [50]. Moreover, a comprehensive marketing mix is established by collaborating with stakeholders to tackle barriers such as accessibility and cost [14].

Social media or mass media and community outreach campaigns [39] were also instrumental at enhancing breast cancer awareness. These campaigns combined broad reach and localized, culturally tailored messaging as well as mitigated specific barriers to care such as stigma and fear. As a result, women were empowered to identify symptoms early and encouraged to visit screening centers [30,39]. However, the actual effect size of changes in breast cancer awareness could not be ascertained given the low-quality research designs. Promising results were also reported in a few qualitative interventional studies involving social media or mass media campaigns [29,37]. Positive advertisements capturing reliable data on breast cancer and endorsements from highly respected celebrities were perceived more positively, leading to proactive health actions. Celebrities can upscale breast cancer awareness and promote positive attitudes toward screening by leveraging their massive reach to destigmatize the cancer, break obstacles, and act as crucial relatable models for early diagnosis [51]. Involving recognizable figures bridge gaps between the general public and complex medical information, translating awareness into life-saving actions [52].

Video-based marketing was primarily used as the source of intervention in 2 studies [18,33], with both reporting greater awareness and improved knowledge and attitudes toward breast cancer screening postintervention. This approach was effective at bypassing literacy barriers, capturing attention, and directly inspiring proactive health behaviors because they rapidly translate complex health information into accessible, emotionally resonant and culturally adaptable formats [53].

Nevertheless, the evidence from the studies needs a deeper assessment and to be interpreted cautiously. Direct comparisons between the interventions was challenging given the different study designs and interventional approaches. However, the large effect sizes reported for SMCs incorporating health promotional activities suggest that these elements are effective at upscaling breast cancer awareness and positive screening decisions. This is further supported by the robust research designs used in the reviewed studies [14]. Community outreach and mass media campaigns also showed promising results in terms of breast cancer awareness and preventive behaviors, with the inclusion of recognizable figures providing additional benefits [54]. Meanwhile, evidence from video-based marketing approaches is weak, and the incorporation of other proven marketing elements may be required to optimize their effects.

Overall, the reviewed studies depict the importance of identifying SMCs and delivery methods that strongly resonate with the target audience [55]. Significant efforts are required to achieve audience segmentation and formative research with the target market for planning and branding the campaign. A catchy slogan and name are critical components of effective SMCs, but the information was lacking in most studies. As a result, it is unclear whether these important elements contributed to the promising outcomes reported in the reviewed studies.

Quality appraisal also revealed that only 2 clinical trials (RCTs) had a robust design. Based on the weak study designs, it is difficult to conclusively infer that the SMCs led to improved breast cancer awareness, screening uptake, and early diagnosis or presentation into care. Although the use of different approaches and evaluation methods provided a clear picture of the population’s exposure to the SMC and the participants’ awareness levels, a standardized approach is required to facilitate robust comparisons between campaigns in terms of effectiveness and economic benefits. We acknowledge the diverse nature of target populations when conducting SMCs, but a standardized reporting of exposure and impact is crucial for significant progress to be achieved in this unique field of public health.

### Limitations

Research into social marketing in public health interventions is rapidly growing, and this scoping review presents only the current and available evidence for social marketing interventions. Only articles written in English were included in our review, thereby neglecting information from relevant interventions either conducted in non-English speaking countries or not written in English. This limitation affects the generalizability of the review findings. Many of the SMCs used combined interventional approaches and sources, possibly contributing to the positive outcomes in terms of breast cancer awareness and screening participation.

Given the lack of a standardized protocol for report evaluations of social marketing interventions, the data synthesis was based on the outcomes measured and reported in the reviewed studies. In other words, some degree of reporting bias might be present in the included studies. The exclusion of gray literature may also introduce some bias, as only articles retrieved from selected databases and peer-reviewed journals were analyzed. The quality appraisal highlighted moderate to weak study designs in 13 of the 15 articles. We did not perform a meta-analysis due to the heterogeneity between the studies, particularly in terms of study designs, delivery methods, type of social marketing interventions, and diverse outcomes. Developing a reporting protocol based on available frameworks for evaluating social marketing research should be considered in future research endeavors.

### Conclusion

This scoping review revealed that behavior change outcomes were sparingly assessed in most evaluations of social marketing interventions to improve breast cancer awareness, screening, and early diagnosis. The limited evidence reflects various types of social marketing interventions, ranging from community outreach to health professional and educational strategies, social media or mass media campaigns, and video-based marketing, which may improve breast cancer awareness and intended and actual screening uptake. Robust evaluations of these interventions could be performed in future research by using well-established evaluation frameworks and reporting guidelines. This will assist with capturing behavior change outcomes and elucidating their underlying mechanisms. Specific users or engaged individuals should be identified, and measures should be used to ascertain whether some health inequalities could be addressed by social marketing interventions. Developers and funders of these campaigns should also consider using an evaluation plan before conducting the actual campaign. Such evaluation plans may consider using feasible mixed methods and quasi-experimental designs. This is crucial to ensuring that campaign exposure and impact are measured adequately, as well as identifying the specific elements responsible for the observed behavioral change.

## Supplementary material

10.2196/88568 Checklist 1 PRISMA-ScR (Preferred Reporting Items for Systematic Reviews and Meta-Analyses Extension for Scoping Reviews) checklist.

## Abbreviations

BCT: behavior change technique
mHealth: mobile health
OR: odds ratio
PEP: Promoting Early Presentation
PRISMA-ScR: Preferred Reporting Items for Systematic Reviews and Meta-Analyses Extension for Scoping Reviews
RCT: randomized controlled trial
SMC: social marketing campaign
